# Biological Response to Carbon-Family Nanomaterials: Interactions at the Nano-Bio Interface

**DOI:** 10.3389/fbioe.2019.00004

**Published:** 2019-01-23

**Authors:** Maryam Rahmati, Masoud Mozafari

**Affiliations:** ^1^Bioengineering Research Group, Nanotechnology and Advanced Materials Department, Materials and Energy Research Center, Tehran, Iran; ^2^Cellular and Molecular Research Center, Iran University of Medical Sciences, Tehran, Iran; ^3^Department of Tissue Engineering & Regenerative Medicine, Faculty of Advanced Technologies in Medicine, Iran University of Medical Sciences, Tehran, Iran

**Keywords:** carbon, carbon nanotubes (CNTs), graphene, fullerene, biomaterials, nanomaterials, nano-bio interface, immunological responses

## Abstract

During the last few decades, several studies have suggested that carbon-based nanomaterials, owing to their unique properties, could act as promising candidates in biomedical engineering application. Wide-ranging research efforts have investigated the cellular and molecular responses to carbon-based nanomaterials at the nano-bio interfaces. In addition, a number of surface functionalization strategies have been introduced to improve their safety profile in the biological environment. The present review discusses the general principles of immunological responses to nanomaterials. Then, it explains essential physico-chemical properties of carbon-familynanomaterials, including carbon nanotubes (CNTs), graphene, fullerene, carbon quantum dots (CDs), diamond-like carbon (DLC), and mesoporous carbon biomaterials (MCNs), which significantly affect the immunological cellular and molecular responses at the nano-bio interface. The discussions also briefly highlight the recent studies that critically investigated the cellular and molecular responses to various carbon-based nanomaterials. It is expected that the most recent perspective strategies for improving the biological responses to carbon-based nanomaterials can revolutionize their functions in emerging biological applications.

## Introduction

Biomaterials science is a multidisciplinary field of study which aims to introduce biological alternatives for biomedical purposes, such as improving tissue and/or organ regeneration (Chen and Liu, 2016; Lee et al., 2018). In today's medicine world, there is an ever-increasing demand for providing promising biomaterials, which could lead to more accurate disease diagnosis and treatment (Calabrese et al., 2015; Karimi et al., 2016). In other hand, nanomedicine is a promising field of study, parallel with other strategies, aims to revolutionize the treatment pathways (Sullivan et al., 2014; Wang et al., 2017). Based on the definition established by National Nanotechnology Initiative (NNI), the field of nanomaterials describes using materials which have at least one length scale ranged between single atomic and one hundred nanometers for fabricating novel systems (Jain and Jain, 2017; Mosayebi et al., 2017; Mozafari et al., 2018). Owing to the well-acknowledged fact that most of the biological components involve some sort of nano-dimensionality, nanomaterials have currently gained an increasing attention among biomedical scientists (Jain and Jain, 2017; Kargozar and Mozafari, 2018). Carbon-based nanomaterials, such as fullerenes, carbon nanotubes (CNTs), and graphene sheets have received considerable attentions among biomedical scientists for multiple emerging applications (Goenka et al., 2014; Bhattacharya et al., 2016; Sivashankari and Prabaharan, 2018). Carbon-based nanomaterials owing to their excellent mechanical, electrical and physical properties, suggest novel promising cues in various fields of biomedicine, such as tissue engineering, delivery systems, and even cancer therapy (Zhang et al., 2014; Shin et al., 2016). As an example, some recent studies have shown that these materials can be potentially employed to fabricate electrically conductive scaffolds with the ability to provide controlled electrical stimulation (Cha et al., 2013; Ahadian et al., 2017). These nanomaterials are promising candidates for developing synthetic bio-scaffolds as suitable platforms in the field of tissue regeneration, as the scaffolds in this area need to precisely mimic the physicochemical and mechanical properties of native extracellular matrix (ECM). It has been commonly acknowledged that carbon-based nanomaterials due to their similar dimensions are physically similar to ECM constituents (Sivashankari and Prabaharan, 2018). In addition, owing to their excellent mechanical behaviors, these nanomaterials play key roles in manipulating biological behaviors. Additionally, the great conductivity of carbon-based nanomaterials could be applied to create or increase the electrical stimulation at the nano-bio interface (Cha et al., 2013).

However, before applying these nanomaterials in the body, their toxicity needs to be meticulously examined. It is known that all the implanted biomaterials could potentially stimulate the immunological responses of body, known as foreign body responses (FBRs), which lead to imperfect functionality in the body (Morais et al., 2010; Trindade et al., 2016). However, FBRs, in other hand, are crucial responses for destroying cellular debris and subsequently inhibiting the infection progression (Gethin, 2012). Therefore, in designing carbon-based nanomaterials carefully considering the details of immune system responses to the implanted material is essential. During implanting carbon-based nanomaterials in the body, the adsorbed proteins, besides macrophages and dendritic cells (DCs) are the key players in starting cellular and molecular communications (Kou and Babensee, 2011). This review emphasis on research concerning carbon-family nanomaterials in biological applications. Here, the general principles of immunological responses to the nanomaterials will be discussed. Then, the main body of the review goes on the essential physicochemical, and mechanical properties of the key carbon nanomaterials including: CNTs, graphene, fullerene, carbon quantum dots (CDs), diamond-like carbon (DLC), and mesoporous carbon biomaterials (MCNs), which significantly impact on the immunological cells responses. The discussion will then highlight the recent studies that deeply investigate the cellular and molecular responses to various carbon nanomaterial surfaces. Finally, the recent strategies which are commonly used for improving the cell responses to carbon-based nanomaterials will be briefly presented.

## Principles Into Immunological Responses to Nanomaterials

**Figure 1:**
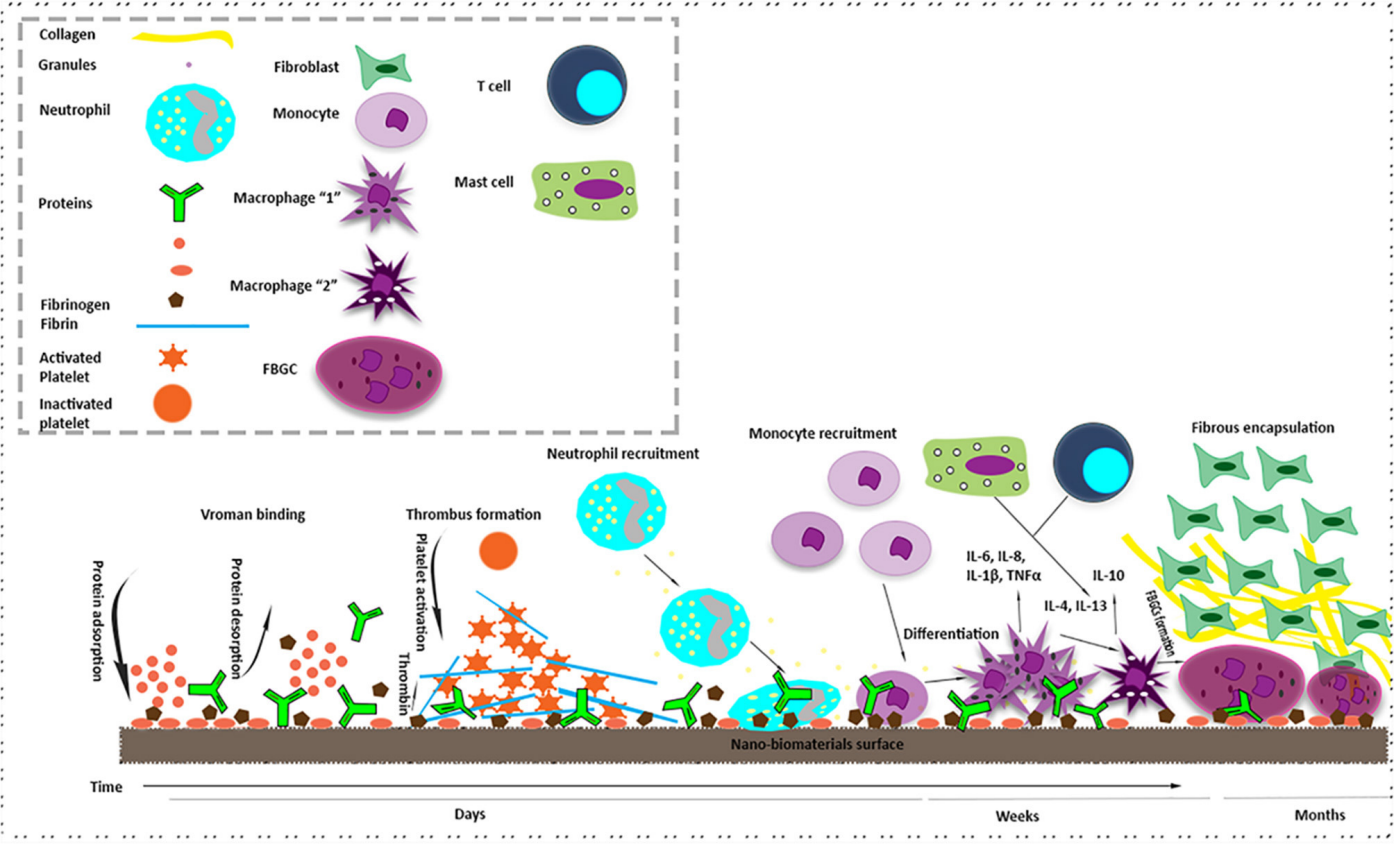
A brief explanation of immunologica l responses at the nano-bio interface (Foreign body response (FBR) to nano-biomaterials). The FBR is a combination of both acute and chronic phases of inflammation. The mechanism starts with protein adsorption and desorption (vroman binding) on the surface of nano-biomaterial after its implantation. It continues with thrombin formation by the activation of platelets. After that, monocytes start differentiation into macrophage type “1” which is responsible for acute phase of inflammation. After some days M1 differentiate macrophage type “2” which is in charge for chronic phase of inflammation. T cells and mast cells also express cytokines that increase foreign body giant cell (FBGC) creation. In addition, FBGCs express Fibroblast recruiting factors and consequently by collagen deposition, a capsule starts forming around the nanomaterial.

Both innate and adaptive immune systems play crucial roles in responding against any implanted biomaterial into the body. In fact, FBRs to the implanted biomaterial potentially define the success or failure of cells-material interactions (Trindade et al., 2016; Rahmati and Mozafari, 2018). It has been demonstrated that the success of implanted nanomaterials differs reliant on the degree of FBRs and following homeostatic mechanism which can potentially lead to the cellular and molecular inflammatory responses. Various humoral and cellular elements are vital to intensify operative immune responses. After nanomaterial's implantation, damaging the blood vessels (as a result of the surgery) causes interactions between the nanomaterial and blood cells through the proteins' adsorption and also provisional matrix formation on and around the nanomaterial's surface (Silva-Bermudez and Rodil, 2013; Wei et al., 2014). The provisional matrix is mainly considered as the primary thrombus/blood clot at the boundary between tissue and nanomaterial, which provokes structural, biochemical, and cellular mechanisms to start regeneration and FBRs processes (Luttikhuizen et al., 2006; Anderson et al., 2008). In addition, during this stage, plasma components, such as proteins, lipids, and ions are rapidly adsorbed on the nanomaterial's surface which start triggering and stopping some mechanisms alongside with the formation of provisional matrix, which finally consequence in the inflammatory responses (Schmidt et al., 2009). After the primary interactions between blood and nanomaterial as well as the development of provisional matrix, acute and chronic inflammation occur in a chronological cascade (Anderson, 2015; Anderson and Jiang, 2017). Stimulated platelets and endothelial cells release chemo-attractants, which consequences in activating neutrophils and acute inflammation phase on the implanted site (Ye et al., 2010; Selders et al., 2017). Neutrophils through phagocytosis and degranulation mechanisms, challenge to eradicate the nanomaterial. Besides, complement proteins alongside with some structures including danger-associated molecular patterns (DAMPs) and pathogen-associated molecular patterns (PAMPs) start the innate immune responses. These patterns are accepted by a restricted amount of pattern recognition receptors (PRRs) which are mainly presented on macrophages and DCs (Esche et al., 2005). DCs are antigen-presenting cells which act as a “bridge” between the innate and adaptive immune system. It has been shown that binding the patterns to PRRs mainly consequences in activating innate immune system through releasing pro-inflammatory cytokines, and chemokines that encourage directed chemotaxis of innate inflammatory cells. It also activates the adaptive immune system through the development of DCs that finally causes B and T lymphocytes initiation (Banchereau and Steinman, 1998; Lanzavecchia and Sallusto, 2001). The adaptive immune system is composed of B cells and T cells, which are highly in charge for immunological “memory” (Iwasaki and Medzhitov, 2015). During the chronic stage of inflammation, the cytokines source is mainly triggered by T lymphocytes, predominantly the T helper (Th) cells which express CD4 and their subsections Th1 and Th2. It has been reported that the cytokine production by these cells highly stimulate both the pro-inflammatory and anti-inflammatory responses (Brodbeck et al., 2005). Additionally, B cells by producing antibodies have a key role in immune system responses to nanomaterials. Besides, it should be noted that lymphocytes could also adhere to nanomaterials surface through pre-adsorbed proteins (Groth et al., 1994). In addition to neutrophils, monocytes also reply to the platelet-derived chemo-attractants placed on the implantation site and make interactions with fibrinogen which finally could cause in their activation (MacEwan et al., 2005; Ward, 2008). The monocytes link to the temporary provisional matrix on the nanomaterial surface through integrins, which play a crucial role in macrophage initiation. These monocytes differentiate into “M1” macrophages at the damage area, which have the talent to release pro-inflammatory cytokines and chemokines (Sheikh et al., 2015; Scatena et al., 2017). It has been shown that the macrophages similar to neutrophils challenge to get rid of nanomaterial, prior to experiencing “frustrated” phagocytosis, eventually causing further pro-inflammatory cytokines activation (Scatena et al., 2017). The activated macrophages finally shift to “M2” phenotype which are recognized by decreased degradative capacity, anti-inflammatory cytokines activation, and starting tissue remodeling process (Vasconcelos et al., 2015). Ultimately, in an effort to develop the phagocytic behavior of macrophages, the formation of foreign body giant cells (FBGCs) on the nanomaterials surface starts, which is mainly initiated via the stimulation of mast cells, basophils, and Th cells (Gordon and Martinez, 2010; Galli et al., 2011; Chung et al., 2017). These cells ultimately release IL-4 and IL-13 which are the key players in creating macrophage fusion (Brodbeck et al., 2002; McNally and Anderson, 2015). A several of studies have shown that macrophages and T lymphocytes stimulated via mature antigen-presenting DCs seem to mainly control the development from chronic inflammation to regeneration. In addition, some studies have shown that mast cells also have a great role in activating pro- and anti-inflammatory, angiogenic and pro-fibrotic factors (Yang et al., 2014). The determined action of immune cells consequences in passageways guided at separating the nanomaterial from the host tissue by fibrotic encapsulation via pro-fibrogenic factors activation, such as platelet-derived growth factor (PDGF), vascular endothelial growth factor (VEGF), and transforming growth factor beta 1 (TGF-beta 1) (Norton et al., 2007; Anderson et al., 2008). In fact, the stimulated fibroblasts accumulate collagen at the implanted site with the aim of repairing the damaged tissue, nevertheless, their unnecessary release consequences in fibrosis (Ward, 2008). If no infection is existent, subsequent to fibrotic encapsulation, the inflammatory reactions eventually will be removed and the implant function leads to tissue regeneration. Therefore, in synthesizing nanomaterials for successfully controlling the immune system responses considering the details of immune cells functions, has a great importance that should be taken into account. A brief explanation of FBRs to nanomaterials surface can be seen in Figure 1. Several of parameters, such as proteins properties, and physicochemical properties of nanomaterials surface potentially impact on the types of adsorbed biomolecules on nanomaterials surface and subsequent responses to it, which regarding carbon-based nanomaterials will be discussed in the next sections.

## The Significant Role of Proteins Adsorption on Cell-Biomaterials Interactions

The adsorption of proteins potentially regulates a series of principal events at the boundary of biomaterial-tissue (Rabe et al., 2011; Wang et al., 2012). In fact, the rapid adsorption of proteins on the biomaterial's surface starts interpreting the surface characteristics into a biological language (Wang et al., 2012). The anchorage and extracellular directions of cells are extremely dependent on the adsorption of some proteins e.g., fibronectin (FN), fibrinogen (Fg), vitronectin, complement C3, and albumin (ALB) (Szott and Horbett, 2011; Wang et al., 2012). Three key driving forces which play crucial roles in the proteins' adsorption on the surface of biomaterials and subsequently cell interactions are including thermodynamically, polarity, and solubility (Dee et al., 2003). It has been reported that thermodynamic, under body environments, could offer a negative free energy charge for proteins' adsorption on the surface of biomaterials (Latour, 2005). Moreover, the proteins' undefined polarity have an influence on their adsorption and prefer a certain level of proteins at boundaries (Anand et al., 2010). Also, there is an opposite connection between protein's adsorption and its solubility (Dee et al., 2003). Some studies have proposed that all bindings between proteins and biomaterial's surface are secondary in nature e.g., hydrogen bonding. In addition, the protein's features that principally affect the surface activities of both biomaterials and cells are related to the key arrangement of the adsorbed proteins and their amino acids' sequence (Barbucci, 2002; Lefèvre et al., 2014). Greater molecules because of their larger surface area are more predictable to interact with surfaces (Latour, 2005). In addition, the hydrophilicity of amino acids, could have influences on the protein adsorption (Vladkova, 2013). Interestingly, the isoelectric point (PI) of proteins could also play a chief role in their adsorption (Kim and Yoon, 2002). Further, by altering the protein's conformation, various amino acids could be presented on the protein's surface, which subsequently change the behavior of adsorbed proteins (Ouberai et al., 2014). The possibility of offering more areas for interacting between protein and biomaterials surface, known as proteins' unfolding, is another key factor, which has an oppose association with the proteins' stability (Dee et al., 2003). The accessibility of proteins for making interaction with the biomaterial's surface should be also considered (Saptarshi et al., 2013). Some studies have reported that the adsorption of proteins on the surface of biomaterials are principally determined through four key transport mechanisms namely diffusion, thermal convection, flow, and coupled transport (Chinn and Slack, 2000). In other side, cells by their specific receptors also interact with the adhesive ligands of ECM proteins, which are also adsorbed on the biomaterial's surface (Dee et al., 2003). The chance of communication between these ligands and cells, is chiefly dependent on the connections of ECM proteins with the biomaterial's surface (Chang and Wang, 2011). After adsorption to the biomaterial's surface, the ECM proteins, owing to their non-rigid formation, experience conformational and orientation alterations which consequently have a great effect on defining the kinds of available ligands for communications with cell surface receptors (Young et al., 1988). Because of the effects of biomaterial's surface properties on the protein's adsorption, the biomaterial properties could directly affect cell responses (Latour, 2005). However, we still suffer from lacking a rational database about the domains involved in this phenomenon and also their precise mechanisms. In overall, precisely defining which intermolecular forces regulate the protein-surface interaction is mostly dependent on the particular protein as well as physical, mechanical, and chemical properties of the biomaterial's surface.

## The Rise of Carbon-Family Nanomaterials: New Roles in Medicine

**Figure 2:**
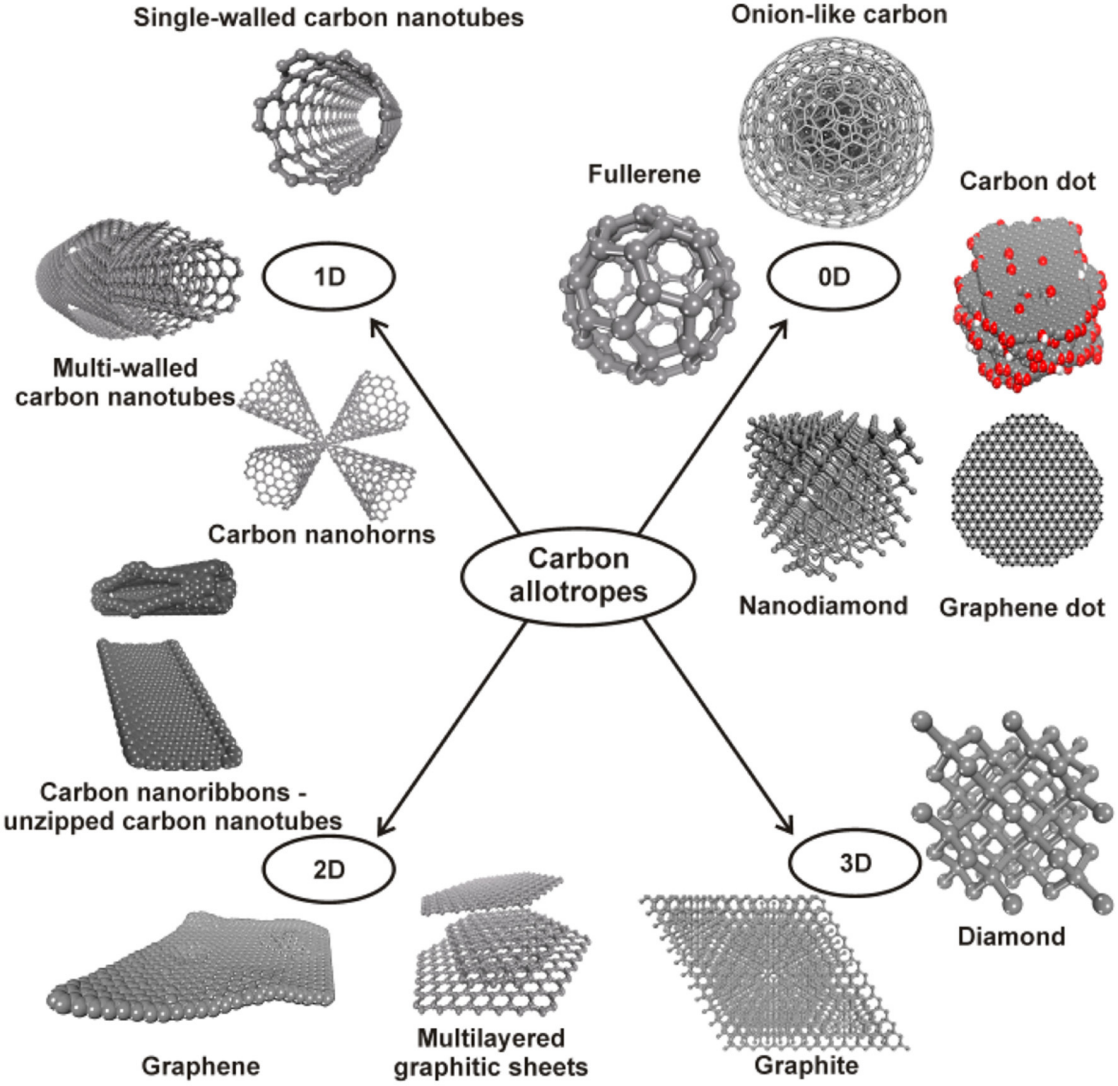
The classification of carbon-based nanomaterials based on their dimensionally. Reprinted from Georgakilas et al. (2015).

In the recent decades, several studies have shown the potential of using carbon-based nanomaterials, such as CNTs (Harrison and Atala, 2007; Touri et al., 2013; Sajid et al., 2016), graphene (Alasv and Mozafari, 2015; Chauhan et al., 2016; Khiabani et al., 2018), fullerene (Goodarzi et al., 2017), QDs (Lim et al., 2015), DLC (Wachesk et al., 2016; Derakhshandeh and Eshraghi, 2018a,b), MCNs (Kim et al., 2008), and CNFs (Yang et al., 2007) in various biomedical applications. The first reports on the Buckminsterfullerene (shortened to fullerene or buckyball) were the first attempts on innovative nano-carbons, leading an extraordinary explosion in universal research (Goodarzi et al., 2017). However, ND which is an important member of carbon nanomaterials has been detected in 1963–1982, C60 generally considered as the earliest detection among symmetric carbon nanomaterials. This discovery further leads to quick progresses in biomaterials field of study (Danilenko, 2006; Goodarzi et al., 2017). Fullerenes and NDs have recently gained a great attention among biomedical scientists, with stress mainly on the field of cancer diagnosis and therapy (Liu et al., 2010; Lichota and Krokosz, 2016). NDs are the products of the famous diamond in nano scale, which are typically recovered from explosion powder. It has been exhibited that via introducing nitrogen positions NDs classically could achieve intrinsic fluorescence (Mochalin et al., 2012). Various strategies, such as diamond nanocrystallites irradiation with helium ions have been suggested for the formation of fluorescent NDs. In addition, CNTs which have two main categorizes including single-walled carbon nanotubes (SWCNTs) and multi-walled carbon nanotubes (MWCNTs), are other key types of carbon nanomaterials family that have gained great attentions among biomedical scientists since their discovery (Harrison and Atala, 2007). SWCNTs are fabricated from graphene sheets which rolled-up to a tube-shaped arrangement, whereas MWCNTs are formed from a number of incorporated CNTs (Du et al., 2013). It has been reported that with the aim of achieving a suitable CNTs for biomedical applications some multifaceted purification techniques are essential (Du et al., 2013). Additionally, graphene, as a single 2-dimensional sheet of carbon, which is the main source of some carbon nanomaterials has been currently reported as a promising candidate in various biomedical applications (Goenka et al., 2014). Some studies have reported the success of applying this kind of carbon in drug delivery systems and tissue regeneration applications owing to its large surface area, exceptional mechanical behavior, and easy functionalization (Goenka et al., 2014; Shin et al., 2016). Probable approaches for graphene fabrication are detaching of single graphene layers from bulk graphite through mechanical or physicochemical exfoliation measures (Novoselov et al., 2004; Geim and Novoselov, 2007). In addition, some studies have reported fabricating graphene by the implementation of surfactants. Chemical Vapor Deposition (CVD) is another method which has been suggested for graphene fabrication (Chen et al., 2011). Graphene oxide and reduced graphene oxide are two derivatives of graphene. In addition, CDs and GQDs, are two other derivatives of graphene which are generally known as small, quasi one-dimensional graphene particles with broad applications in bio-imaging (Xue et al., 2011; Li et al., 2013; Dong et al., 2014; Dasari Shareena et al., 2018; Yarahmadi et al., 2018). These derivatives could be fabricated by the implementation of some approaches including the carving of graphene, direct wet chemical routes, and hydrothermal techniques (Loh et al., 2010; Xu et al., 2010). The structure of different types of carbon-based nanomaterials could be observed in Figure 2. Recently, biomedical scientists have done many efforts with the aim of modifying the cellular and molecular responses to carbon nanomaterials via using many strategies for carbon nanomaterials surface modification. It has been widely reported that if the biocompatibility issue of carbon nanomaterials, which still remains a big challenge in biomaterials field of study, solved these nanomaterials owing to their exceptional physicochemical properties could suggest some promising diagnostics and treatments clues in medicine.

## Impact of Physicochemical Properties on Cellular and Molecular Responses

**Figure 3:**
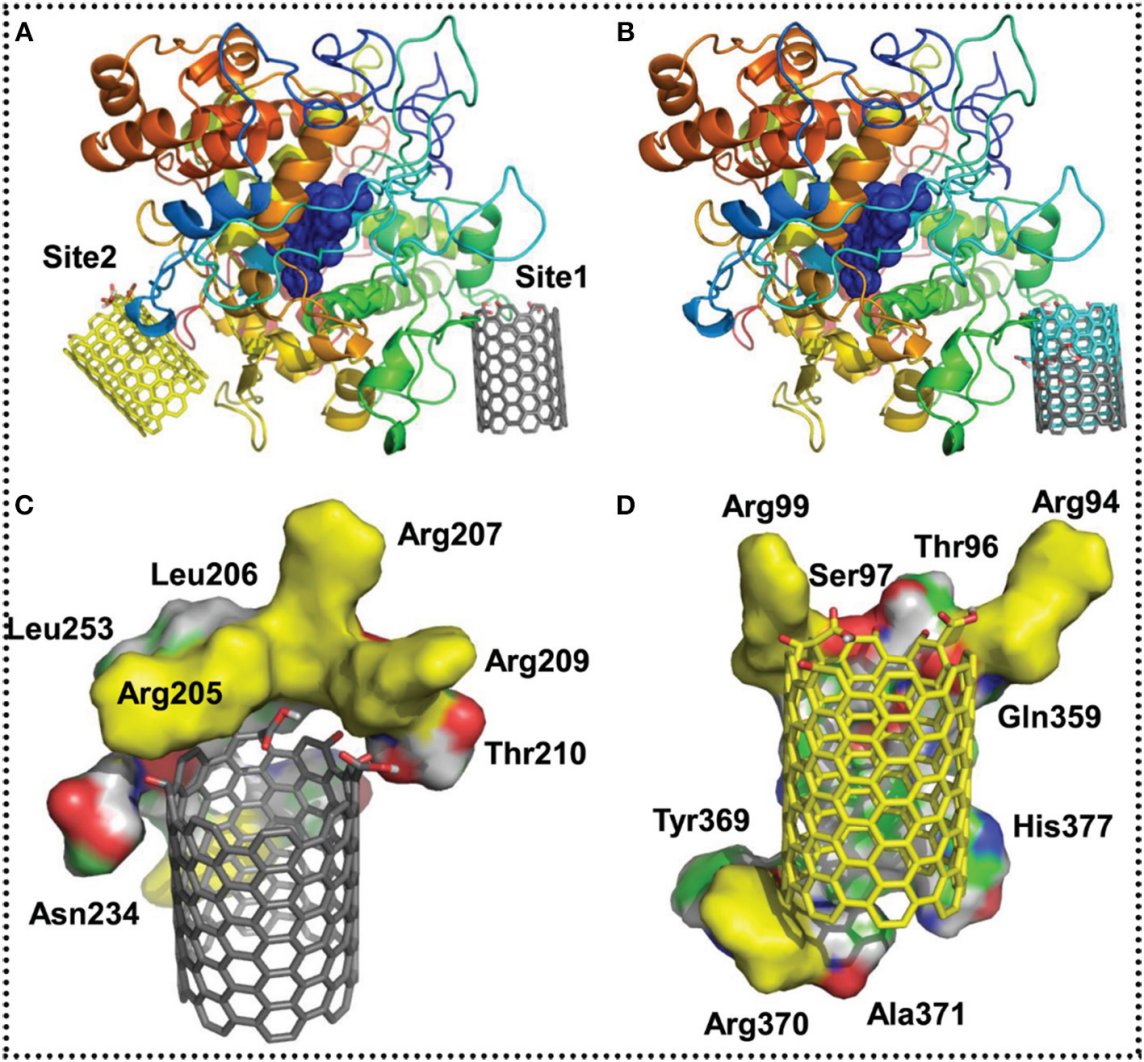
Molecular modeling of probable SWCNTs interaction positions on eosinophil peroxidase (EPO). **(A)** The two expected interaction places, position 1 and 2 of oxidized SWCNTs improved at the edge. The oxidized SWCNTs matching to position 1 and 2 are rendered as sticks and colored in gray and yellow, correspondingly. **(B)** A connection of the probable interaction position 1 of SWCNTs oxidized at the edge (colored in gray) and in the middle (colored in cyan). The remainders that are in close nearness (with in 4 Å), stabilizing the binding sites **(C)** position 1 and **(D)** 2. Positively charged remains (arginines) that are expected to steady the oxidized groups on SWCNTs are colored in yellow. The network of EPO is colored in rainbow from N to C terminus in **(A,B)**. Reprinted from Andón et al. (2013) with the permission from Elsevier.

It is a well-known fact that the physicochemical properties of biomaterials could have significant effects on the molecular and cellular responses (Roach et al., 2007; Mitragotri and Lahann, 2009). There have been several attempts to explore the potential effects of the physicochemical properties of carbon-based nanomaterial's surface on the protein adsorption and subsequently cell responses. However, it should be taken into consideration that because of the limitations related to precisely analyzing the surface topography, the evaluation of results is often challenging. Further, the techniques for engineering physical properties of biomaterials' surfaces could also have an influence on their chemistry properties. Some studies have demonstrated that the topographical surface properties of carbon-based nanomaterials could regulate the cells behaviors in two ways including; directly through affecting cytoskeleton or indirectly via protein alignment and unfolding (Lim and Donahue, 2007; Fraczek-Szczypta et al., 2015; Park and Im, 2015). It has been reported that the surface roughness could potentially increase the hydrophobicity of carbon-based nanomaterials' surface (Deng et al., 2015). In addition, the size of carbon-based nanomaterials could have a meaningful impact on the molecular and cellular responses (Fujita et al., 2015). The shape of carbon-based nanomaterials could also noticeably affect the cellular responses (Bacchetta et al., 2018). Electrostatic interactions also play a role in the biocompatibility of carbon-based nanomaterials owing to the fact that all the boundaries in fluid solutions are charged, and also the cell membranes have negative charges (Yang et al., 2007; Klingeler and Sim, 2011). Electrically conducting carbons which their surface properties could be significantly changed by applying an electrical potential, have been broadly offered as promising candidates for biological applications (Gomez-Gualdron et al., 2011; Mehra et al., 2015; Sajid et al., 2016). The mechanical properties of carbon-based nanomaterials could potentially impact on cells as they response in contradiction of the biomaterial's surface and send their reactions to the nucleus about the surroundings (Baradaran et al., 2014). In other hands, the chemical properties of carbon-based nanomaterial's surface, known as chemisorption, are highly important in determining the chemical or covalent interactions between the surface and cells. Some studies have been doing on investigating the impact of surface chemical functionality on cell responses (Dumortier et al., 2006). In fact, several of studies have manipulated the cellular responses to the carbon-based nanomaterials by chemically modifying their surfaces. Surfaces carrying functional groups with different hydrophobicity and charges have been demonstrated to have impact on the cell responses. Current investigations have demonstrated that besides hydrophobicity/hydrophility, the surface functional groups also highly impact on protein and cell responses (Srivastava et al., 2017). The degradation properties of carbon-based nanomaterials could also have effects on the molecular and cellular responses. The biological degradability of carbon nanomaterials is still a challenge despite their broad applications. It has been shown that some carbon-based nanomaterials could demonstrate asbestos-like pathogenicity, in part owing to the fiber-like morphology, and also the hypothesis that CNTs are biopersistent. Although, some researchers have suggested some methods for increasing the biodegradability of these materials by considering the role of innate immune system in the enzymatic digestion (Kotchey et al., 2013; Bhattacharya et al., 2016). However, chemical degradation of these materials has been exhibited by either harsh chemical treatment with mineral acids140 or degredation of graphitic lattices via inducing high temperatures, these methods could not be effective in a biological system (Li et al., 2008; Liu L. et al., 2008). It has been currently shown that peroxidases which have robust redox potentials, are recognized to catalyze oxidation of foreign particles and pathogens with hydrogen peroxide in the body. In addition, the catalytic heme active site is a representative of mammalian peroxidases, such as neutrophil myeloperoxidase (MPO), eosinophil peroxidase (EPO), and lactoperoxidase (LPO), which have been demonstrated to catalyze the degradation of carboxylated SWCNTs by producing reactive radical intermediates (Andón et al., 2013) (Figure 3).

**Figure 4:**
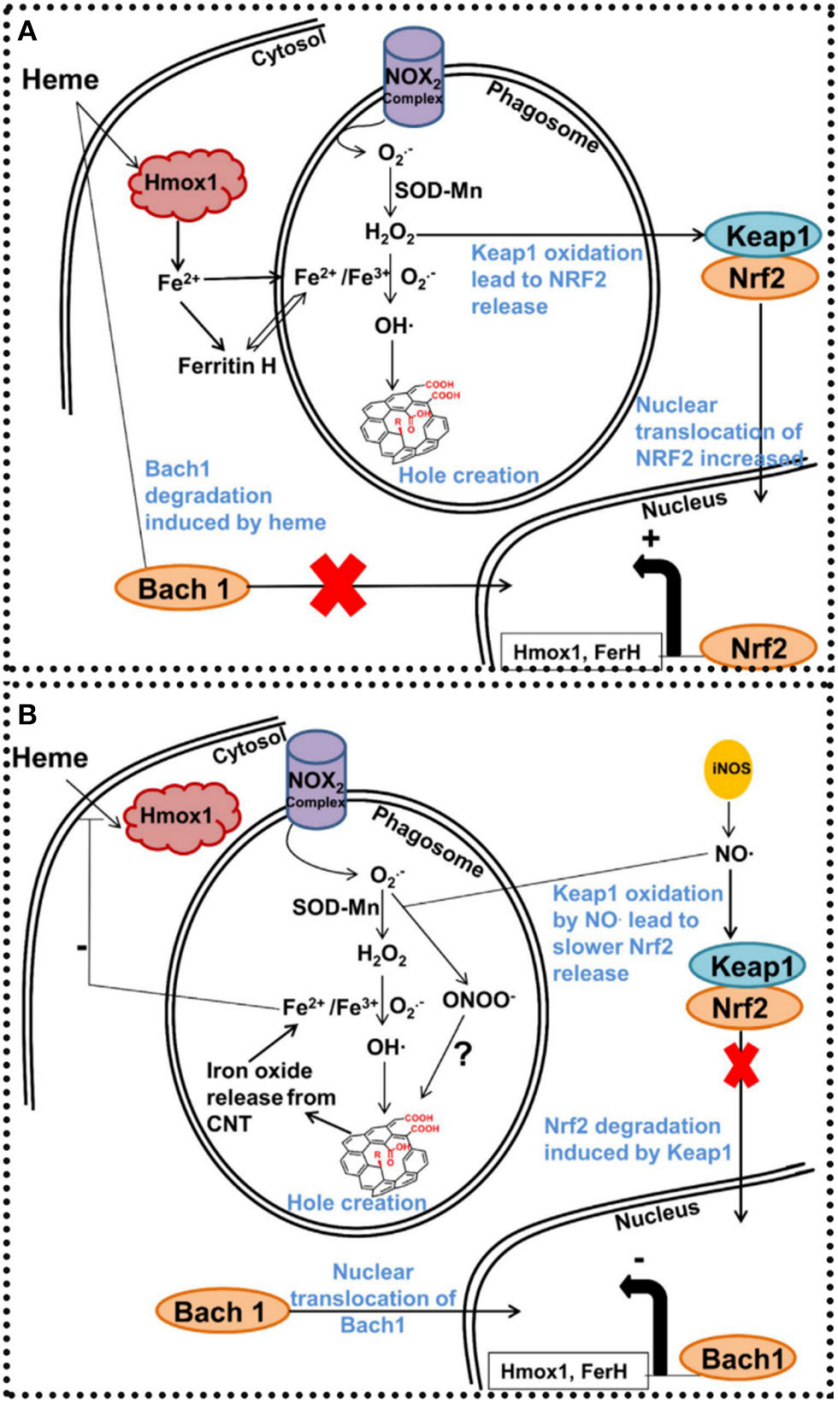
Schematic depiction of the iron's role in MWCNT degradation mechanism in macrophages. After phagocytosis of CNTs, NOX_2_ complex is stimulated both on cytosolic and phagosomal membranes. Active NOX_2_ complex encouraged O_2_ production. O_2_ is turned into hydrogen proxide by SOD into phagosome, and hydrogen proxide is turned into hydroxide in the presence of Fe^3+^ via the Haber-Weiss mechanism. After that, hydroxide radicals attack CNTs to produce carboxylic acids that form holes in the graphitic arrangement. **(A)** In the lack of iron encapsulated into the CNT network, iron employed for Haber-Weiss reaction has to be produced by the cells. Consequently, oxidative stress products like hydrogen proxide encourage Keap1 cysteine oxidation and free Nrf2 for nuclear translocation. FerH and Hmox1 proteins, will be interpreted for iron creation. **(B)** In the existence of iron filled CNTs, iron from xenobiotics are changed into Fe^2+^ and Fe^3+^ in the acidic environment of the phagosome. Excess of iron ions prevent heme entry in the cells and consequently encourage Bach1 nuclear translocation and FerH and Hmox1 repression. As iNOS was encouraged after Fe@MWCNT contact, it is probable that Keap1 cysteine will be oxidized via NO. Reproduced from Elgrabli et al. (2015).

In addition, Elgrabli et al. (2015) have currently studied a molecular pathway which through that macrophages degraded functionalized MWCNTs considered for biological applications with having, or not, iron oxide nanoparticles in their internal cavity. Their results indicated that intracellularly-induced network defects appear more quickly for iron-free CNTs compared with iron-loaded ones, which showed the possible role of iron in the degradation mechanism. They compared the molecular responses of macrophages to both types of CNTs, and exhibited a molecular mechanism controlled by Nrf2/Bach1 signaling pathways to encourage CNT degradation via NADPH oxidase 2 (NOX_2_) complex activation and O_2_, H_2_O_2_ and OH production (Figure 4). CNT exposure stimulated an oxidative stress-dependent production of iron with Nrf_2_ nuclear translocation, Ferritin H and Heme oxygenase 1 translation. Contrariwise, Bach1 was translocated to the nucleus of cells unprotected to iron-loaded CNTs to recycle embedded iron. In overall, their outcomes suggested new data on the role of oxidative stress, iron metabolism and Nrf2-mediated host defense for manipulating CNT fate in macrophages (Elgrabli et al., 2015).

## Carbon-Family Nanomaterials At The Nano-Bio Interface

Although carbon-based nanomaterials have shown exceptional physicochemical properties that make them suitable candidates for biomedical applications, due to their possible toxic behavior, their clinical usage still remains challenging. Some studies have been done on evaluating the biocompatibility of these nanomaterials for biomedical applications. In addition, several of studies have more currently focused on providing promising surface modification strategies to enhance the biocompatibility of carbon-based nanomaterials. There is a crucial need in the literature for separating possible destructive carbon nanomaterials from safe ones. In biomedical fields of study, it is essential to not make a general view about the toxicity of carbon nanomaterials, because each class of them has various physicochemical properties which directly impact on their biocompatibility profile. Therefore, through carefully assessment of the cellular and molecular responses to each type of carbon nanomaterials a further promising scenario for their biomedical utilization will be provided. For these reasons, in the next sections, the physicochemical properties of each type of carbon-based nanomaterials which potentially impact on the cellular and molecular responses will be discussed in detail.

### Cellular and Molecular Responses to Fullerenes

**Figure 5:**
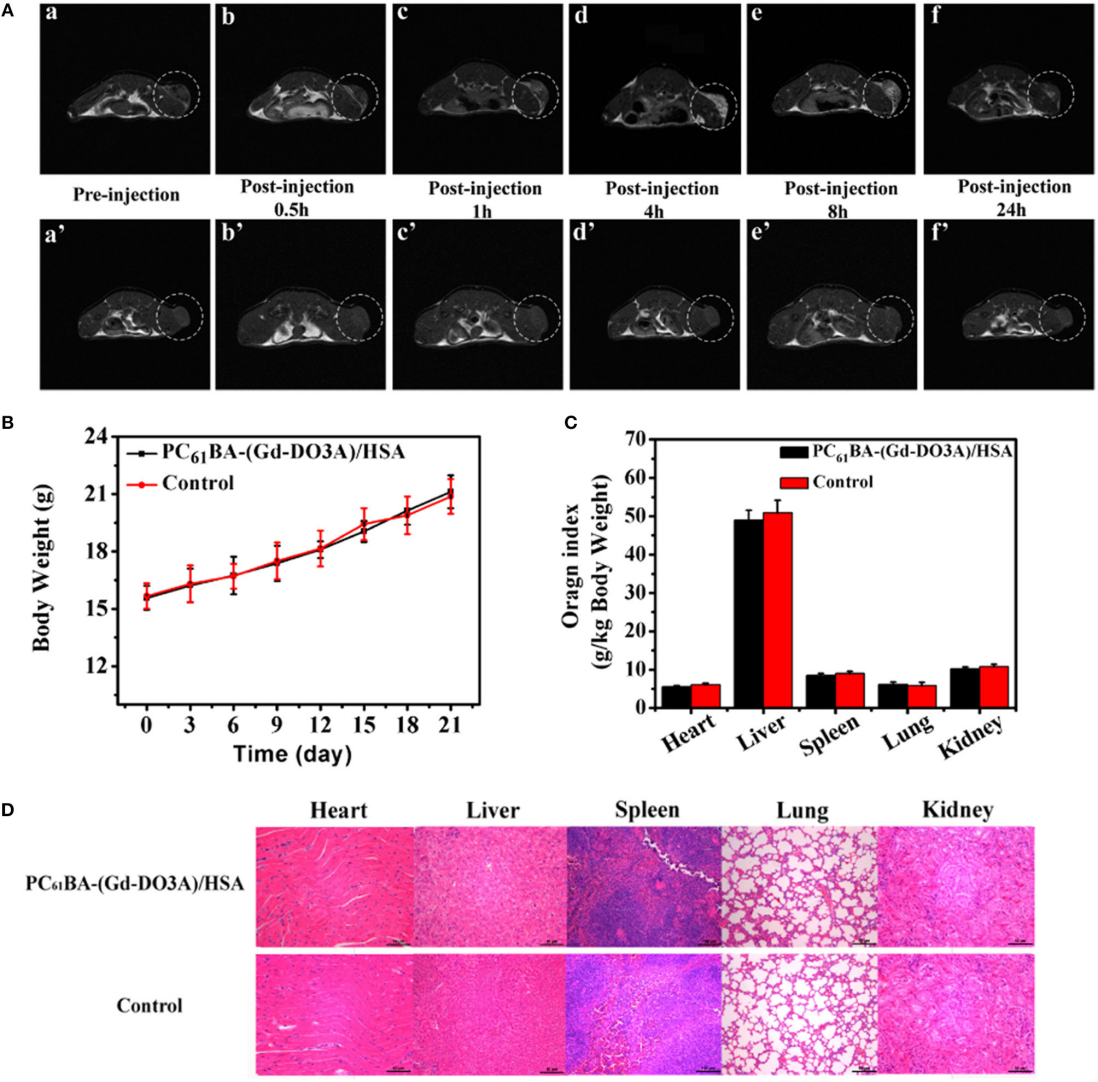
**(A)** T1-weighted MRIs of tumor-bearing BALB/C mice before (a) and after injecting PC61BA-(Gd-DO3A)/HSA (0.04 mmol Gd3+/kg bw) at 0.5 (b), 1 (c), 4 (d), 8 (e), and 24 h (f) and those of tumor-bearing BALB/C mice before injection (a′) and after injection of Gd-DO3A (0.04 mmol Gd3+/kg bw) at 0.5 (b′), 1 (c′), 4 (d′), 8 (e′), and 24 h (f′). The tumor tissue is painted in the white dotted ring. **(B)** Changes in the bw of mice which were injected with the agent or saline **(C)** Evaluation of organ directories of the mice treated with both presented agent and saline as control sample. **(D)** histologiacl studies of mice injected with the suggested agent (above) and saline (bottom) as control. Adopted from Zhang et al. (2016) with the Elsevier Permission.

Fullerene as a carbon-based nanomaterial which possesses a typical cage arrangement, has been suggested as a promising candidate for biomedical applications (Da Ros and Prato, 1999; Goodarzi et al., 2017). Since this class of materials at the beginning has been introduced for medical purposes, its biocompatibility assessments were examined at the time of its discovery. Some researchers have investigated the macrophages responses to non-treated fullerene, CNTs, and graphite and then concluded that the cytotoxicity of fullerene was lesser than that of CNTs and graphite (Jia et al., 2005). Some studies have suggested that the concentration of fullerene has an influence on its biological responses. Tolkachov et al. (2016) have recently studied the viability of human mesenchymal stem cells (hMSC), and HeLa, as a cancer cell line, on fullerene surfaces by MTT test, which their results indicated that C60 could encourage the growth of both cell types at low concentrations (6–12 μg/mL) and cause their viability reduction at high concentrations (24 μg/mL). Sayes et al. (2004) have examined the cytotoxicity of some different fullerene classes, and determined that variations in its cage arrangement had influences on its cytotoxicity behavior. It has been exhibited that water-soluble fullerene showed substantial cytotoxicity to cultured cells, however an extremely hydroxylated, water-soluble fullerene, exhibited no indication of cytotoxicity under the similar circumstances (Panessa-Warren et al., 2006). Additionally, some studies have examined the biocompatibility of C60 by exposing it to different cultured cells and concluded that fullerene cytotoxicity was because of lipid peroxidation of the cell membranes. Besides, some researchers have suggested that the cytotoxicity of fullerene derivatives is influenced by the ligands (Oberdörster, 2004). Also, the residuals of tetrahydrofuran (THF) which is employed for improving fullerene purification and dispersion could increase its cytotoxicity (Kovochich et al., 2009). Zhang et al. (2016) have recently introduced a novel magnetic resonance imaging (MRI) contrast mediator by conjugating the gadolinium/1,4,7,10-tetraazacyclododecane-1,4,7-tetracetic acid complex (Gd-DO3A) with 6,6-phenyl-C61 butyric acid (PC61BA) and human serum albumin (HSA). As it can be observed in Figure 5, they showed that the suggested mediator, had a great constancy and showed a considerable upper relaxivity than Gd-DO3A under the same condition, which indicated the efficacy of using HSA and fullerene for improving the relaxivity of Gd-DO3A. The *in vivo* MR images of on tumor-bearing mice demonstrated great signal improvement for the tumor site owing to the developed penetrability and retention effect. The highest amassing of PC61BA-(Gd-DO3A)/HSA at the tumor position was accomplished at 4 h after injection, which could direct surgery. Additionally, the hematological and histological studies revealed no clear toxicity of injected treated-samples *in vivo*, which all together indicated the potential efficacy of using the proposed agent for tumor diagnosis. It should be noted that there is a contradiction between studies have been done on fullerene's biocompatibility, which needs much further meticulous *in vitro* and *in vivo* investigations.

### Cellular and Molecular Responses to CNTs

**Figure 6:**
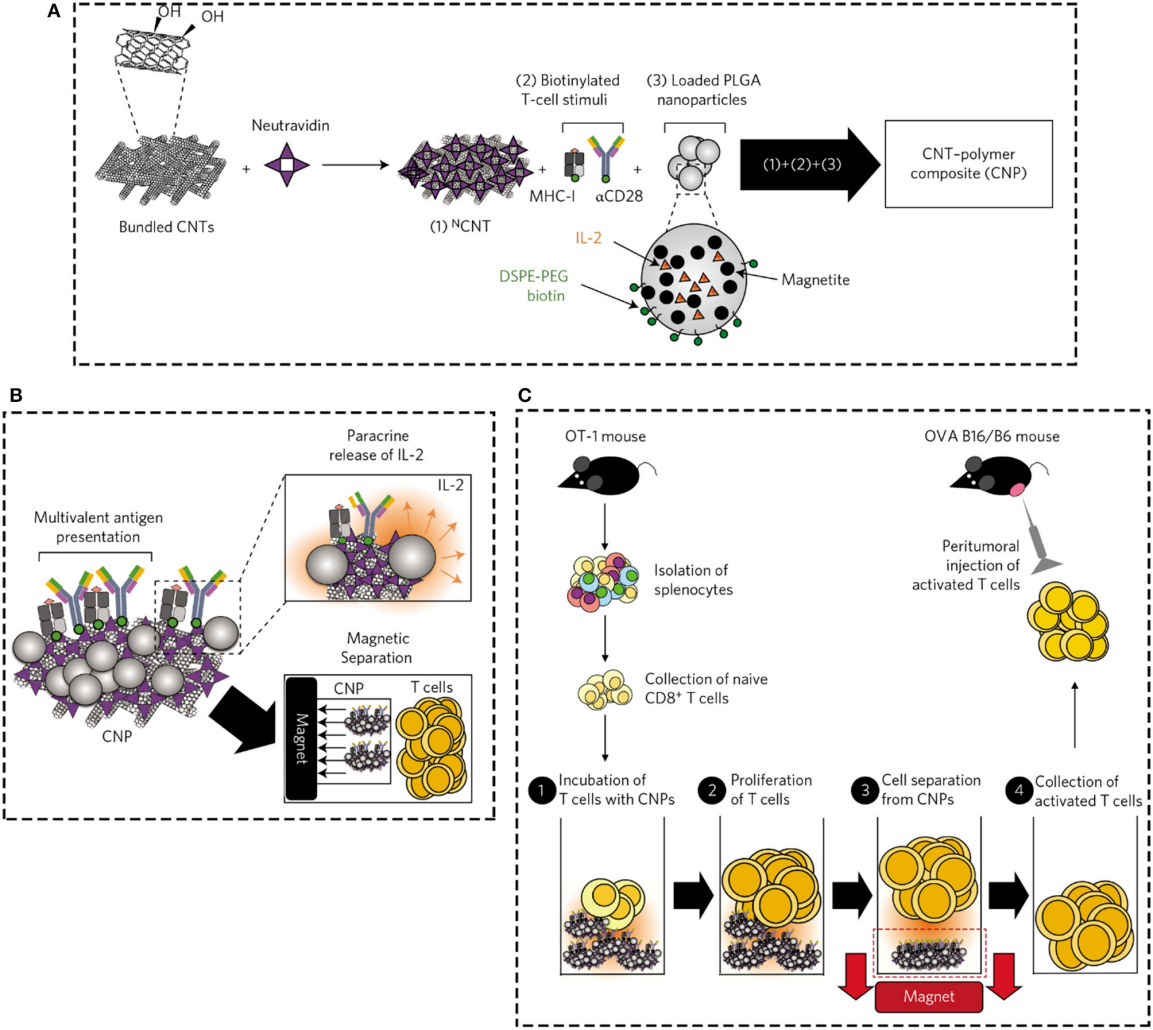
**(A)** The adsorption of neutravidin as a protein linker onto the CNTs to cause eutravidin-bound CNTs (^N^CNTs), and also connection of the biotinylated T-cell stimulatory signals to the surface. **(B)** Through binding PLGA NPs containing IL-2 and magnetite to ^N^CNTs, the authors combined the multivalent demonstration of biological T-cell stimuli with paracrine delivery of IL-2, whereas facilitating the magnetic departure of CNPs from T cells. **(C)** After magnetic departure from CNPs, the beneficial efficiency of those stimulated T cells in mice protected melanoma cells that stimulate the secretion of B16-OVA. Reproduced from with Fadel et al. (2014) Elsevier permission.

The physicochemical properties, such as size, shape, specific surface area, wall number, size distribution, and chemical composition of CNTs are highly responsible for immunological system responses to them (Smart et al., 2006; Du et al., 2013; Touri et al., 2013; Xue, 2017). It has been shown that nanoparticles in two ways could pass into cells including directly via the cell membrane or indirectly via infiltrating in the space between cells, which then translocate to the blood circulation and diffuse all through the body (Geiser et al., 2005). Therefore, nanoparticle's size is a key parameter which by suggesting larger surface area to the substrate highly impacts on the translocation potential, FBRs, distribution and elimination of nanoparticle at the cellular and molecular level (Powers et al., 2006). It has been commonly accepted that greater surface area increases the availability of possible positions for making connection with proteins and cells. Some studies have reported that the physicochemical possessions of dispersion medium, as well as particle aggregation and agglomeration could to a great extent affect the size of nanoparticles. Agglomeration and aggregation are the attractions that cause amassing the nanoparticles. More precisely, the agglomeration of nanoparticles is the formation of particles clusters which try to hold together by electrostatic interactions, while aggregates are shaped from covalently bonded or sintered nanoparticles, which could not simply detach them (Maynard et al., 2006; Johnston et al., 2010). CNTs owing to their electrostatic attractions have a great tendency to create bundle or rope arrangements, which make it difficult to evaluate their biocompatibility in the body. Some studies have suggested using dispersing agents, solvents, surface attachments, and mechanical procedures for improving the CNTs dispersion which following that enhance their biocompatibility (Johnston et al., 2010; Alshehri and Ilyas AM, 2016). It has been reported that the shorter SWCNTs with larger surface area, more easily carried the proteins and oligonucleotides into the body cells than bigger ones (Kam et al., 2006). Additionally, the smaller CNTs have the chance to be uptake by a broader range of cells and also translocated over various cellular barriers (Kostarelos et al., 2007). Some studies have suggested that MWCNTs owing to aggregation and agglomeration phenomena were harder phagocytized by macrophages and transported to local lymph nodes than SWCNTs, which could provoke more cytotoxic effects in the body (Fraczek et al., 2008; Yang et al., 2008). In addition, some studies have reported that the length of CNTs could strongly effect on fiber clearance, due to the fact that it provokes the talent of phagocytic cells to totally internalize CNTs. The longer fibers increase the frustrated phagocytosis, and decrease clearance, which finally cause increasing their tendency to damage (Johnston et al., 2010). Brown et al. (2007) have reported that MWCNT with longer length had the ability to increase the secretion of pro-inflammatory cytokines, and finally provoke frustrated phagocytosis phenomenon. Furthermore, a several of studies have proved that different shapes of nanomaterials including planes, spheres, tubes, rings and fibers could also have effect on cellular and molecular responses. Haniu and his coworkers (Haniu et al., 2014) have more currently investigated the effects of the shape and size of MWCNTs and cup-stacked carbon nanotubes (CSCNTs) on human bronchial epithelial (BEAS-2B) and malignant pleural mesothelioma cells responses. In this study, three kinds of MWCNTs including VGCF®-X, VGCF®-S, and VGCF® (vapor grown carbon fibers; with diameters of 15, 80, and 150 nm, correspondingly)—and 3 CSCNTs of dissimilar lengths (CS-L, 20–80 μm; CS-S, 0.5–20 μm; and CS-M, of intermediate length) were examined. Their results exhibited that CSCNTs were fewer toxic than MWCNTs in both cell lines. The biocompatibility of endocytosed MWCNTs were different based on cell type/size, where as that of CSCNTs was governed by tube length regardless of cell category. In addition, the diameter and length of CNT affected cell aggregation and injury degree. In overall, they concluded that CSCNTs could be appropriate for biomedical applications and also that CNT shape and size could potentially have different impacts reliant on the cell type (Haniu et al., 2014). Verma and Stellacci (2010) have currently reported that the shape of CNTs could influence the absorption and desorption of biomolecules on the surface of them and also the membrane warping process throughout endocytosis or phagocytosis. It has been demonstrated that CNTs with the tubular arrangement, have the ability to definitely get through capillaries and stick to blood vessel (Radomski et al., 2005). In addition, CNTs with the tubular arrangement could provoke the accumulation of platelets and following that speed up the vascular thrombosis rate in animal model (Radomski et al., 2005). Moreover, the tubular shape of carbon nanomaterials could make them able to block potassium ion channels (Johnston et al., 2010). In addition, some studies have revealed that nanoparticles surface charge (cationic, anionic, or neutral) by influencing on their size scatterings, translocation, shape and agglomeration plays a key role in cellular and molecular responses (Hoshino et al., 2004). The surface charge of nanomaterials strongly affects protein adsorption and desorption on nanomaterials surfaces. In general, a several of studies have reported that nanomaterials with cationic surfaces by provoking complement activation, organism hemolysis, and platelet aggregation are further toxic than anionic, and neutral ones (Goodman et al., 2004; Mayer et al., 2009). Additionally, the roughness of carbon nanomaterials surfaces plays a significant role in their interactions with biological systems (Nel et al., 2009). Apart from physical properties of CNTs surfaces, their surface chemistry also is a key factor affecting cellular and molecular responses (Kirchner et al., 2005). By using various surface modifications strategies and the existence of different contaminants, CNTs could have dissimilar chemical composition. Bardi et al. (2013) have currently examined the potential interactions between chemically functionalized MWCNTs and the neural tissue after cortical stereotactic administration. They fabricated two kinds of f-MWCNTs including shortened (via oxidation) amino-MWCNT as well as amino-f-MWCNT, then evaluated their effects on neural cells responses. The authors found that both types of treated MWCNTs were up-taken by microglia, astrocytes and neurons cells, however, their cellular internalization patterns were dissimilar. Also, both types of treated MWCNTs provoke inflammatory cytokines secretion. Although, the oxidation of amino-MWCNT encourage more substantial amounts of GFAP and CD11b expression nearby to the *f*-MWNT injection site. It has been reported that residual metal catalysts (such as iron and nickel), amorphous carbon and hydrocarbons are possible impurities which mainly presented throughout CNTs production. Some studies have exhibited that the presence of these metal impurities in CNTs potentially decrease their biocompatibility (Harper et al., 2008; Kostarelos et al., 2009). In the recent decade, some strategies, such as acid and heat treatments have been used to increase the CNTs purity, however, these approaches could negatively affect the CNTs arrangement and subsequently their biological responses (Raja et al., 2007). Some studies have deeply investigated the effects of using different surface chemistry modifications on cell responses to CNTs. It has been demonstrated that nitrogen-doped MWCNTs were more biocompatible and adsorb further proteins than pure MWCNTs. Moreover, modification of CNTs with carboxyl group is one of the commonly used approaches to improve CNTs water dispersibility. Although there is a scarcity of data in the literature regarding their cytotoxicity effects on biological conditions. Liu et al. (2014) have currently studied the effects of carboxylated MWCNT on human normal liver cell line (L02) in comparison with pristine MWCNT. Their results indicated that both p-MWCNT and MWCNT-COOH, at definite concentrations, encouraged meaningfully reducing the mitochondrial membrane potential, and increasing the release of cytochrome c from mitochondria to cytoplasm as well as activation of caspase-9, and -3. The authors found that functionalization of MWCNT surfaces with carboxyl group decreased the toxicity of MWCNT on L02 cells, which was potentially owing to reducing the activation of mitochondria mediated apoptotic mechanism. Some research groups have suggested using the moieties, such as proteins for improving the efficiency of macromolecules delivery more efficiently to cells (Karajanagi et al., 2004). The current role of employing novel modification strategies for improving surface chemistry of carbon nanomaterials including CNTs which directly affect cellular and molecular responses will be discussed later in the presented paper. Fadel et al. (2014) have more recently suggested a novel carbon nanotube–polymer composite that acts as a synthetic antigen-presenting cell to powerfully enlarge the T cells numbers. The authors adhered antigens onto hydroxyl-treated bundled CNTs which had a high degree of surface defects and also a high surface area, then joined the system with PLGA nanoparticles comprising magnetite and the T-cell growth factor interleukin-2 (IL-2). As it can be seen in Figure 6A, neutravidin as a protein linker was adsorbed onto CNTs to cause neutravidin-hydroxyl-treated bundled CNTs (NCNTs), then the biotinylated T-cell stimulatory signals were connected to the CNTs surfaces. After that, PLGA nanoparticles comprising magnetite and the T-cell growth factor interleukin-2 (IL-2) were combined with the CNTs, which through that they combined the multivalent presentation of biological T-cell stimuli with paracrine IL-2 delivery, however, the CNTs were separated from T cells via encapsulated magnetic (Figure 6B). Following that, after magnetic departure from CNPs (Figure 6C) they examined the beneficial efficiency of those stimulated T cells in mice protected melanoma cells that stimulate the secretion of ovalbumin antigen (B16-OVA). In overall, their results indicated that the proposed system could be a promising substrate for significantly increasing the proliferation and function of cytotoxic T cells for cancer immunotherapy.

### Cellular and Molecular Responses to Graphene-Based Nanomaterials

Graphene is a one-atom-thick, 2D planar sheet with trigonal bonded sp^2^ carbon atoms, which are strongly arranged into a honeycomb crystal framework (Kuila et al., 2012; Alasv and Mozafari, 2015). Graphene has a large surface area which makes it a suitable candidate for delivery systems. In fact, the large surface area of graphene makes it possible that every atom reaches its surface, and is uncovered to the neighboring medium on both sides of this material (Geim and Novoselov, 2007; Loh et al., 2010). It has been suggested that the large surface area of graphene nanomaterials, particularly monolayer graphene and graphene oxide, have a great influence on their cellular and molecular responses. Some studies have revealed that their large surface area caused provoking reactive oxygen species (ROS) production or quenching, and antioxidant deactivation. However, it has been reported that graphene nanomaterials, owing to their hydrophobic nature, have a tendency to aggregate in salted surroundings, such as phosphate buffered saline (PBS) and protein rich cell culture medium, which hinder their use as a delivery system (Liu Z. et al., 2008; Liao et al., 2011; Chowdhury et al., 2014). It has been suggested that by using the oxidized form of graphene (known as graphene oxide), the aggregation difficulty of graphene could be to a great extent solved. Graphene oxide has an analogous arrangement to pristine graphene with a variability of chemically reactive functionalities, which has a great hydrophobic basal plane and hydrophilic edges (Lerf et al., 1998; Loh et al., 2010). Graphene oxide by possessing some advantages over CNTs, such as amphiphilic structure, larger surface area, higher drug loading capacity and lower amount of metallic contaminations in its composition, has gained a great attention among biomedical scientists for drug delivery applications (Liu Z. et al., 2008; Bussy et al., 2012; Zhang et al., 2012). Additionally, graphene oxide owing to the ionization of the carboxylic acid and hydroxyl groups has a more stable dispersion than CNTs in water solutions (Li et al., 2008). Moreover, graphene layers' number which affects the particular surface area and bending stiffness is another key factor which should be taken into consideration. Some investigations have demonstrated that the protein adsorption will meaningfully improve on the surface of graphene as the layer number reduces. However, the precise role of stiffness in the biological responses to graphene as a plate-like material has not yet been explained (Patra et al., 2009; Bellido and Seminario, 2010; Guo et al., 2011; Sanchez et al., 2011). Additionally, lateral dimension is another key parameter which by defining the maximum dimension of the graphene, affect the cellular uptake and other biological responses to it (Sanchez et al., 2011). It has been reported that lateral dimension of materials could have an influence on the amount of receptors which are essential for cellular uptake and also the size of endosome or lysosomes which are in charge for packaging foreign materials. It has been suggested that lateral dimension of graphene could also affect the deformability of it, so that laterally large graphene is further deformable than small ones at identical layer number (Sanchez et al., 2011). In addition to physical properties of graphene, its surface chemistry could also have a great influence on its biological behavior (Gao, 2015). The graphene-based nanomaterials have a broad different surface chemistry structures. For instances, as above mentioned, graphene oxide has an amphiphilic structure with partly hydrophobic and hydrophilic areas capable of hydrogen connection and metal ion complexing, and encompass carboxylate negative groups on edge sites (Cote et al., 2008; Hsieh and Chen, 2011). However, the original graphene has a hydrophobic surface, which mainly starts interactions with biomolecules at the edge or defect sites. Reduced graphene oxide, in other hand, has medium hydrophilic domains on its surface structure (Bagri et al., 2010). In addition, however, graphene nanomaterials do not encompass residual metal catalysts, some of them could enclose residual intercalants, which are chemical additives applied to distinct the layers in the bulk graphite feedstock. Moreover, during the fabrication of graphene oxide some reagents are used which could subsequently leave some soluble residues in the suspension if they are not correctly washed (Kim et al., 2010). Wang et al. (2013) have recently studied FBRs to various physicochemical properties of both graphene and MWCNTs. They found that after iv administration of GNS to the lungs of mice models, it stimulated a Th2 immune response at first day, which included increasing the concentration of neutrophils, IL-5, IL-13, IL-33, and its soluble receptor (sST2) in the broncho-alveolar lavage fluid. However, MWCNT provoked a meaningful growth in the messenger ribonucleic acid expression of cytokines in the spleen containing IL-4 and IL-33, which were directly connected with a rise in CD4^+^ and CD8^+^ T-cells expression in spleen. Their histological tests demonstrated that some agglomerated MWCNT amassed in the pulmonary capillary at first day, which did not spread in the alveolar spaces, whereas graphene evidently touched the alveolar spaces at the same time point. The contradictions between results could be explained by considering the differences between physicochemical conditions of these carbon-based nanomaterials, as graphene with its flat form, seem to effortlessly enter the endothelial barrier of the pulmonary capillary. However, MWCNT, with its tubular formation effortlessly agglomerate and could not simply enter the endothelial barrier, which result in hindering their entry into the lung. In addition, as Cell autophagy is a significant immunological system response to foreign nanomaterials, Wan et al. (2013) have more currently investigated the special effects of AF-SWCNTs and graphene oxides on cell viability, autophagy induction and lysosome destabilization. Their autophagosome, lysosome, as well as p62 protein degradation results indicated that both AF-SWCNTs and graphene oxides provoked the adverse effects in AF-macrophages cells, however, graphene oxide was further effective than AF-SWCNTs. Additionally, in another study which has been done by Park et al. (2015), it has been more clearly demonstrated that the commercially available graphene nanoplatelets could highly induce a sub-chronic inflammatory response in mice models and also autophagy alongside with apoptosis through mitochondria injury *in vitro*.

**Figure 7:**
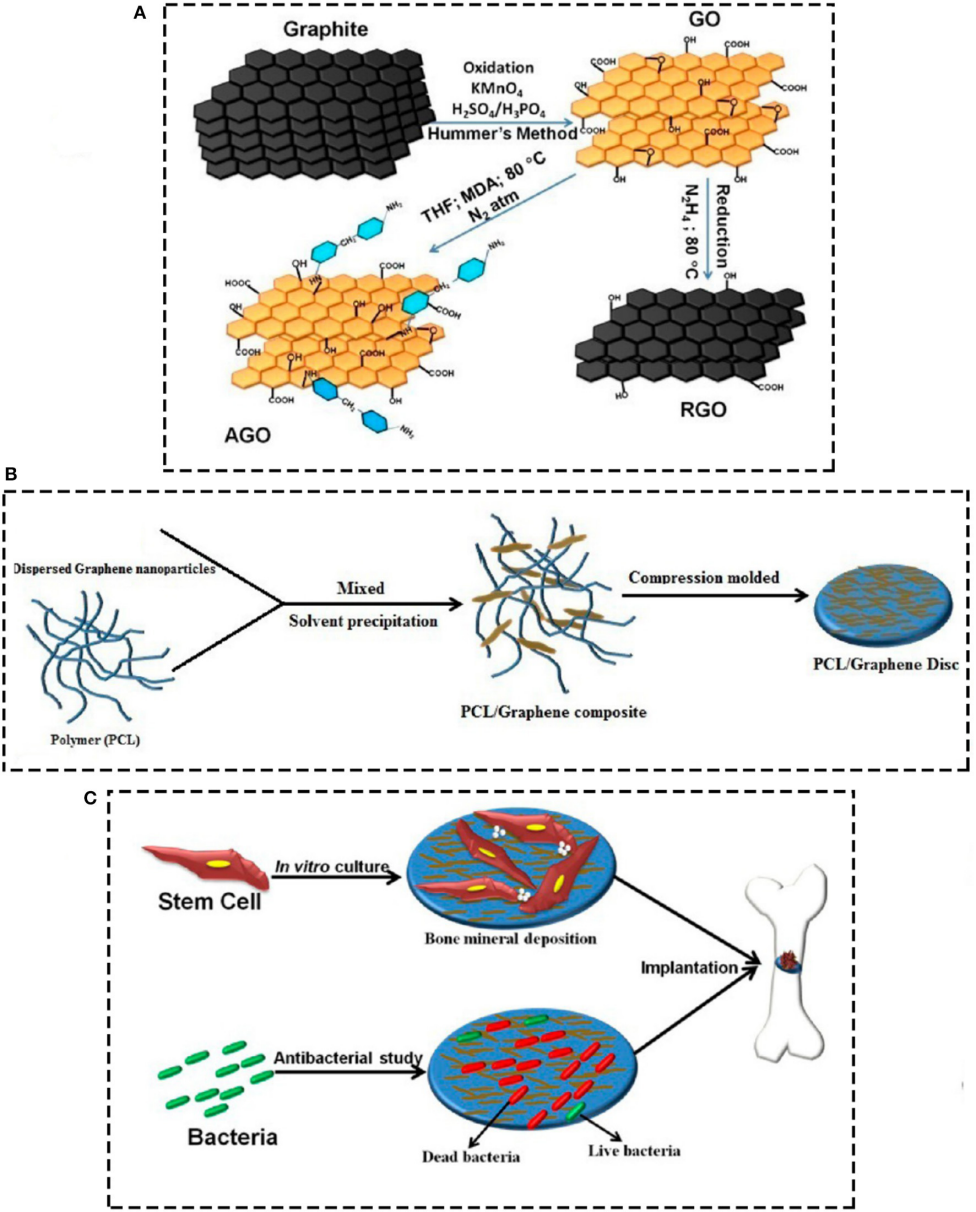
Schematic demonstration of **(A)** fabricating graphene derivative nanoparticles (GO, RGO, and AGO), GO was made by chemical oxidation of natural graphite flakes subsequent with Hummers technique, RGO was made from GO by chemical reduction approach **(B)** fabricating PCL/graphene scaffolds by solvent precipitation approach, and **(C)** study of stem cell and bacterial response to the substrates for orthopedic applications. Reprinted with the permission from Kumar et al. (2015). Copyright 2018 American Chemical Society.

Kumar et al. (2015) have currently studied the efficacy of using PCL-based scaffolds containing different kinds of chemical functionalized graphene oxides for orthopeadic applications. As it can be seen in Figure 7, they synthesized various PCL scaffolds containing graphene oxide, reduced graphene oxide, amine-functionalized graphene oxide (AGO) via solvent precipitation approach, and then the effects of any of the functionalization methods on mechanical properties, stem cell response, and biofilm formation were assessed. They detected that by adding graphene nanomaterials the storage modulus of PCL scaffolds increased, with the greatest increase for reduced and amine-functionalized graphene oxides. In addition, regarding cell viability studies, graphene oxide and amine-functionalized graphene oxide nanoparticles could highly stimulate the stem cell proliferation, however, the AGOs were more operative in expanding stem cell osteogenesis which led to mineralization. Furthermore, the bacterial examinations indicated increasing bacterial cell death by exposing to functionalized graphene oxides, with the highest rate of death and prohibiting biofilm formation for amine-functionalized substrates. It should be taken into consideration that there is a contradiction between studies have been done on graphene's biocompatibility, which needs much further meticulous *in vitro* and *in vivo* investigations.

### Cellular and Molecular Responses to DLC Surfaces

DLC surfaces have a diversity of properties, such as great hardness, near to the ground friction coefficients, chemical inertness, great resistance to wear, and good biocompatibilities, which make them promising candidates for biomedical applications (Allen et al., 1994; Butter and Lettington, 1995; Wei et al., 2016; Derakhshandeh et al., 2017; Huacho et al., 2017). DLC films are promising applicants for modifying the surface of artificial joints, which the cell responses to its surface properties have attracted attentions of scientists in recent years (Liao et al., 2016). Many techniques are suggested to fabricate the DLC films including ion beam assisted deposition, sputtering, cathodic arc, pulse laser ablation, and PECVD (Spencer et al., 1976; Ahmed et al., 2015; Wei et al., 2016). It has been reported that DLC that was fabricated by dual beam ion technique did not show any cytotoxicity after exposing to macrophages and mouse fibroblasts. In addition, Allen et al. (1994) have made DLC by using plasma-activated CVD and demonstrated that the murine macrophage cell line had a good viability profile on its surface. Moreover, in another study it has been exhibited that human synovial fibroblasts and human “osteoblast-like” cells had a well-growth on DLC substrate with no sign of abnormal morphology. Butter and Lettington (Butter and Lettington, 1995) have investigated the efficacy of using DLC-coated pins into soft tissue and femurs of sheep, which their results indicated much superior bonding at DLC than metal–tissue borders with inferior risk of infection. Huacho et al. (2017) have more recently studied the surface free energy (SFE), wetting and surface properties, antimicrobial, adhesion and biocompatibility characteristics of DLC surfaces for biomedical applications. Their results indicated that the DLC surfaces cause a small reduction in cell viability, whereas the SFE, roughness (R a), bacterial adhesion, antimicrobial, and bacterial infiltration investigations presented no noteworthy dissimilarities. They concluded that the DLC is a biocompatible material with slight cytotoxicity which did not illustrate variations in *R* a, SFE, bacterial adhesion or antimicrobial properties and also did not hinder the permeation of *E. coli* into the abutment-dental implant boundary. Liao et al. (2016) have recently investigated the cellular responses to DLC films with dissimilar properties by using macrophages, osteoblasts and fibroblasts. Their results indicated that DLC films with a low ratio of sp2/sp3, had fewer inflammatory responses indicating by lesser expression of TNF-α, IL-6, and upper expression of IL-10, with exceptional osteogenic and fibroblastic responses. Additionally, this type of DLC films exhibited better BSA adsorption without electrostatic repulsion. Moreover, it has been currently demonstrated that DLC films comprise of sp2/sp3 hybrid non-crystalline carbon nano-particles with the size of about 50 nm could meaningfully decrease the platelet adhesion to implants and subsequently to a great extent improve their hemo-compatibility (Wei et al., 2016). Ahmed et al. (2015) have currently doped silicon on the surface of DLC films and then studied the impacts of DLC surface morphology on its interaction with HAS. The authors reported that the films containing silicon by increasing the surface roughness of DLC substrates improved the adsorption level of HAS. Miksovsky et al. (2014) have currently examined the human osteosarcoma cells responses to thin ultrananocrystalline diamond (UNCD) and DLC films with various surface modifications including O_2_ or NH_3_/N_2_ plasmas and UV/O_3_ treatments. Their results showed that none of the treatment approaches caused an alteration of the surface topography; however, they caused a surface composition alteration by increasing amounts of oxygen and nitrogen. The chemical composition changes led to increasing the hidrophilicity of substrates, with better conditions for UNCD films. In addition, the surface energy of modified samples significantly increased. Increasing the hydrophilicity and surface energy of surfaces led to enhancing cell responses to the substrates, especially in the case of UNCD films.

### Cellular and Molecular Responses to Carbon-Based Dots

Carbon-based dots, containing graphene quantum dots (GQDs), and carbon nanodots (CDs), are a novel formula of 0D carbon-based nanomaterials (Hsu et al., 2012; Liu and Chen, 2013; Lin et al., 2014). So far, a several of papers have been published in the area of carbon-based dots and a substantial development has been accomplished in the fabrication and various applications of them (Li et al., 2012; Philippidis et al., 2013; Miao et al., 2015). GQDs are graphene frameworks that typically have fewer than ten layers of graphene (Zhang et al., 2012). These carbon-based dots are exceptional electron presenters and acceptors, which could be applied in fabricating photodetectors and solar cells, electrochemical biosensors (Gupta et al., 2011; Zhao et al., 2011). It has been demonstrated that directing the graphene's layers could cause change from GQDs to CDs structures, which are quasi-spherical carbon nanomaterials with the diameter of fewer than 10 nm. The internal portion of CDs is generally contained *sp2* hybridized carbon atoms, whereas its external portion comprises *sp3* hybridized carbon atoms (Yu et al., 2012). C, H, N, O are the main elements of CDs, which C and O are the greatest presented elements owing to the existence of carboxylic acid moieties. It has been suggested that the presence of these groups offered an exceptional water solubility, and also increased the opportunity for additional modification with various strategies. Some studies have revealed that CDs have many advantages including robust Photoluminescence (PL) emission in observable spectral range, exceptional water solubility, low toxicity, confrontation to photo bleaching, simplicity of production and surface functionalization, which all make it a favorable candidate for some biomedical applications, such as biosensing, bioimaging, and drug delivery (Liu and Chen, 2013; Yuan et al., 2014; Miao et al., 2015).

### Cellular and Molecular Responses to MCNs

In the recent years, biocompatible inorganic mesoporous materials have gained a great consideration among biomedical scientists owing to their high surface area, pore capacity, and adjustable pore sizes which offer great substrates for biomolecules (Chen et al., 2009, 2013; Ambrogio et al., 2011). One of the well-known mesoporous materials in the field of biomaterials is mesoporous silica nanoparticle (MSN) which possesses high biocompatibility, controllable biodegradation and sustained drug-releasing profile with simple surface functionalization (Wu et al., 2011; Shi et al., 2013; Singh et al., 2016, 2017). Mesoporous carbon nanoparticles (MCNs) are another class of 0D mesoporous biomaterials which have been hardly suggested for biomedical applications, possibly because of the deficiency of suitable synthetic procedures to formulate MCNs with required physiochemical properties for biomedical applications (Chen and Shi, 2015). It has been shown that the MCNs which are synthesized by the implementation of traditional nano-casting approaches have asymmetrical morphology with high particulate size. Additionally, these type of carbons are naturally hydrophobic, which strictly limits their possible biomedical applications. However, MCNs with sphere-shaped morphology could more ease the free transport of encapsulated drugs or biomolecules within blood vessels in comparison with other carbon-based nanomaterials. In addition, these materials demonstrated good biocompatibility, sustained drug-releasing profile, simple surface functionalization, high photo-thermal-conversion efficacy, with some exceptional theranostic activities. Hence, if the mentioned difficulties of this type of carbon-based nanomaterials are elucidated they could be promising candidates for biomedical applications (Chen and Shi, 2015). Some studies have suggested employing oxidization approaches by using strong acids to enhance the hydrophilicity of MCNs (Zhu et al., 2012). It has been reported that this kind of modification could also present different functional groups on the surface of these materials.

## Surface Modification for Improving Biological Responses

As above mentioned, the propensity of CNTs to aggregate in large bundles and ropes is a difficulty which makes it hard to manipulate the materials for improving their biocompatibility and subsequently characterize them (Tagmatarchis and Prato, 2004). In fact, it has been suggested that the high percentage of disagreements in toxicity statistics could be owing to the dissimilarities in CNT dispersion. Some studies have recently provided some strategies for improving CNTs solubility such as; sonication, stabilization with surfactant and covalent functionalization. Sonication is a generally employed approach which could without adding any chemical modification rapidly disperse CNT aggregates in solution. The ultrasonic bath and the ultrasonic probe are two key approaches of sonication, which use a bubble nucleation and collapse device (Hilding et al., 2003). However, because after using sonication approach still some degrees of CNTs aggregates were detected in aqueous solution, a great attention has been recently paid to organic synthetic surfactants which are commercially accessible, and cost-effective with simple preparation procedures (Kam and Dai, 2005; Muller et al., 2005; Smart et al., 2006). Sodium dodecyl sulfate (SDS), TritonTM X-100, and PluronicTM are the main surfactants that are used for improving carbon nanomaterials solubility (Moore et al., 2003; Lin et al., 2004). Biological and bioactive species, such as DNA, carbohydrates and proteins are frequently considered as surfactants to solubilize carbon nanomaterials in aqueous solution (Barisci et al., 2004; Yang et al., 2007; Karousis et al., 2010). It has been exhibited that these kinds of modifications make it possible to more precisely assess the cellular and molecular responses to the nanomaterials. In addition, the progress of using controllable modifications approaches via employing these biological components provides further promising solutions in the direction of applying carbon-based nanomaterials in different biomedical applications. In the recent years, by discovering the important role of carbohydrates in living system a new type of coating (known as sugar-coated) has been introduced as a promising coating method for these nanomaterials (Fahrenholtz et al., 2015). In addition, some studies have suggested that nucleic acids could be successfully coated on the surface of carbon-baes nanomaterials and following that improve its biocompatibility (Singh et al., 2006; Apartsin et al., 2014). Additionally, functionalization of carbon surfaces through proteins, such as enzymes and antibodies is another interesting methods of surface modification (Mehra et al., 2014; Kim et al., 2017). However, some preparation circumstances have been detected that cause surfactant detachment from CNTs surface (Lin et al., 2004). Chemical functionalization of carbon nanomaterials is another universally used strategy for enhancing solubility (Tagmatarchis and Prato, 2004). This approach by using covalently attaching suitable molecules, such as peptides, acids, amines, polymers and poly-L-lysine to the carbon nanomaterials surfaces improve their solubility to a great extent (Smart et al., 2006). Some studies have reported that the chemical functionalization of carbon nanomaterials or adsorption of biomolecules is the best method for improving the carbon nanomaterials solubility for biomedical applications (Cherukuri et al., 2004; Shi Kam et al., 2004). Burleson et al. (2009) have modified the NDs in different acid, base, and organic solutions under hydro- and solvo-thermal environments in order to investigate the impacts of adding different functional groups onto the surface of NDs on their biocompatibility. They concluded that adding functional groups on the surface of NDs is highly dependent on the solvent and process factors. In addition, adding CO, OH, or NH functional groups could highly improve the biocompatibility of NDs (Burleson et al., 2009). Additionally, Kabur and his colleagues have reported that the dispersion of fullerene in water can be significantly enhanced by adding sulphuric acid on its surface. Moreover, as some studies have reported the sever thrombogenicity of graphene oxide, Singh et al. (2012) have recently studied the effects of chemical functionalization of this nanomaterial with NH_2_ on platelet responses. After *in vivo* studies on mice models, they found that the modified graphene oxides did not stimulate platelets responses, pulmonary thromboembolism, and oerythrocytes lysis (Singh et al., 2012).

## State-of-the-Art and Future Prospective

As above described, carbon-based nanomaterials have exceptional physicochemical properties which could be highly useful in different biomedical applications, such as bio-imaging, delivery systems, and biosensors. However, due to the scarcity of *in vitro* and *in vivo* data in literature regarding their safety issue in the biological conditions, there is a crucial need to establish their biocompatibility prior to their clinical usage. Some more *in vitro* and *in vivo* investigations should be also done on the long-term effects of carbon nanomaterials on lung toxicity. The contradiction between the obtained results of published papers in the literature indicates that still much work should be done in establishing the safety of carbon-based nanomaterials (Smart et al., 2006). However, some *in vivo* studies in this regard could be seen in the literature, many of them have been done on mice models, which owing to the tremendous differences between immunological system of human and mice, the achieved results should be taken into account with a high caution (Shay et al., 2013; Orecchioni et al., 2014). In addition, there is a scarcity of information in the literature regarding the cytotoxicity effects of carbon nanomaterials on the skin tissue and cells. Additionally, as it has been reported that immunological cells had an inflammatory response to these materials, the precise FBRs to them should be also taken into consideration (Jia et al., 2005). Furthermore, as carbon-based materials have different methods of preparation with various degrees of impurities, during investigating their biocompatibility in biological systems considering every detail of their physicochemical properties is of great importance. Moreover, the non-biodegradability of carbon-based nanomaterials is another issue which should be taken into consideration (Foldvari and Bagonluri, 2008). Owing to the crucial effects of physicochemical properties of carbon nanomaterials on their biological manner in future studies, better standardization of materials characterization should be taken into account. In addition, studying the long-term efficacy of each functionalization method on carbon biological manners and their potential risks should be more precisely examined. A several of studies have exhibited that using DLC coatings on biomaterials surfaces provided satisfactory circumstance for the growth of cells without any sign of cytotoxicity (Roy and Lee, 2007). In addition, DLC coatings meaningfully reduce the wear and corrosion and subsequently releasing metal ions from orthopedic implants. However, it should be noted that DLC coating demonstrates a broad range of atomic bond arrangement and properties dependent on the deposition situation. Moreover, the adhesion of DLC coating to implants should be more precisely investigated (Roy and Lee, 2007). Furthermore, regarding graphene oxide, some simple approaches for reproducible synthesis and meaningful batch-to-batch characterization of it, should be provided to more precisely control its physicochemical properties. Some studies should also be done on determining the optimal safe dosage of graphene for biological applications. It has been also suggested that owing to the high propensity of graphene oxide to accumulate in lungs, developing its design for lung disorders should not be ignored (Bussy et al., 2012; Kuila et al., 2012; Alasv and Mozafari, 2015). Besides, some studies have revealed that MCNs owing to containing the mixture of mesoporous nanostructure and carbon structure, as well as high biocompatibility, could be considered as the next generation of inorganic biomaterial systems for biological usages. However, further studies is essential to improve the clinical usage of MCNs (Chen and Shi, 2015). In addition, the analytical apparatuses for revealing and characterization of CNTs should be more technologically advanced, as there is now a difficulty of studying the precise interactions between biomolecules with carbon nanomaterials. In overall, as there is a huge contradiction between studies that have been done on the safety of carbon-based nanomaterials for biological applications, far more understanding of this paradox is essential. By considering this contradiction, it is too early to conclude anything about the safe using of carbon-based nanomaterials in tissue-engineered scaffolds.

## Conclusion

In summary, here we have reviewed the current research developments of using carbon-based nanomaterials in biomedical applications by improving their interactions at the nano-bio interface. A vast number of papers have been published in the recent years which suggested some promising approaches for achieving better biological responses to these types of nanomaterials. However, additional development and utilization are still instantly anticipated. There is a paradox between studies that have investigated the biocompatibility of carbon nanomaterials due to various kinds of preparation and modification approaches, which finally lead to have very dissimilar physicochemical properties. To sum up, the main problems regarding using these materials are their slow or non-degradability as well as particle aggregation and agglomeration, which should be solved before any biological usage. In addition, there is a crucial need for long-term *in vitro* and *in vivo* investigation of cellular and molecular responses to each physicochemical property of these nanomaterials. The use of carbon nanomaterials for various biomedical applications, such as bioimaging, drug delivery, and biosensing provides a challenging but hypothetically worthwhile chance to progress the next generation of engineered nanomaterials.

## Author Contributions

MR wrote the first draft. MM revised the text and finalized the draft.

### Conflict of Interest Statement

The authors declare that the research was conducted in the absence of any commercial or financial relationships that could be construed as a potential conflict of interest.

## Abbreviations

NNI: national nanotechnology initiative
CNTs: carbon nanotubes
ECM: extracellular matrix
FBRs: foreign body responses
DCs: dendritic cells
CNTs: carbon nanotubes
CDs: carbon quantum dots
DLC: diamond-like carbon
DAMPs: danger-associated molecular patterns
PAMPs: pathogen-associated molecular patterns
PRRs: pattern recognition receptors
FBGCs: foreign body giant cells
Th: T helper
PDGF: platelet-derived growth factor
VEGF: vascular endothelial growth factor
TGF-beta 1: transforming growth factor beta 1
SWCNTs: single-walled carbon nanotubes
MWCNTs: multi-walled carbon nanotubes
CVD: chemical vapor deposition
CSCNTs: cup-stacked carbon nanotubes
AGO: amine-functionalized GO
BEAS-2B: human bronchial epithelial
PBS: phosphate buffered saline
hMSC: human mesenchymal stem cells
R a: roughness
THF: tetrahydrofuran
MPO: neutrophil myeloperoxidase
EPO: eosinophil peroxidase
LPO: lactoperoxidase.
